# Adaptation Aftereffects in Vocal Emotion Perception Elicited by Expressive Faces and Voices

**DOI:** 10.1371/journal.pone.0081691

**Published:** 2013-11-13

**Authors:** Verena G. Skuk, Stefan R. Schweinberger

**Affiliations:** 1 Department for General Psychology and Cognitive Neuroscience,Institute of Psychology, Friedrich Schiller University of Jena, Jena, Thuringia, Germany; 2 DFG Research Unit Person PerceptionInstitute of Psychology, Friedrich Schiller University of Jena, Jena, Thuringia, Germany; University of British Columbia, Canada

## Abstract

The perception of emotions is often suggested to be multimodal in nature, and bimodal as compared to unimodal (auditory or visual) presentation of emotional stimuli can lead to superior emotion recognition. In previous studies, contrastive aftereffects in emotion perception caused by perceptual adaptation have been shown for faces and for auditory affective vocalization, when adaptors were of the same modality. By contrast, crossmodal aftereffects in the perception of emotional vocalizations have not been demonstrated yet. In three experiments we investigated the influence of emotional voice as well as dynamic facial video adaptors on the perception of emotion-ambiguous voices morphed on an angry-to-happy continuum. Contrastive aftereffects were found for unimodal (voice) adaptation conditions, in that test voices were perceived as happier after adaptation to angry voices, and vice versa. Bimodal (voice + dynamic face) adaptors tended to elicit larger contrastive aftereffects. Importantly, crossmodal (dynamic face) adaptors also elicited substantial aftereffects in male, but not in female participants. Our results (1) support the idea of contrastive processing of emotions (2), show for the first time crossmodal adaptation effects under certain conditions, consistent with the idea that emotion processing is multimodal in nature, and (3) suggest gender differences in the sensory integration of facial and vocal emotional stimuli.

## Introduction

The perception of emotional states is crucial for adequate social interaction. Emotions are expressed in the face, but also in the voice (e.g., [1]), or in gesture (e.g., [2]), and body movement (e.g., [2-4]). Although the majority of empirical studies investigated emotion perception in one modality only, many researchers now think that emotions are perceived in a multimodal manner [5]. Evidence supporting this idea includes reports on brain-damaged patients, who showed comparable impairments in processing specific emotions from faces and voices (e.g., [6,7], but see also 8).

An impressive source of evidence for the perceptual integration of facial movements and speech is the so called McGurk effect [9], which shows that simultaneous presentation of an auditory vocalization with non-matching facial speech can alter the perceived utterance (e.g. the presentation of an auditory /baba/ with a face simultaneously articulating /gaga/ typically leads to a “fused” percept of /dada/). A possible neurophysiological correlate of this effect has been described in studies that show an activation of auditory cortex when participants watched silent facial speech, in the absence of an auditory stimulus [10]. However, crossmodal processing is much less well investigated for paralinguistic social signals, including person identity and emotional expression (for a recent overview, see 11).

One of the first studies to report audio-visual integration in emotion perception was by de Gelder and Vroomen [12], who showed that the presentation of sad or happy voices with an emotion-ambiguous test face biased perceived facial emotion in the direction of the simultaneously presented tone of voice, even when voices should be ignored. Similar findings for facilitated processing of emotion-congruent audio-visual emotional signals were found by others [13,14]. More recently, evidence from magnetoencephalography has suggested that the posterior superior temporal sulcus area may be involved in the early perceptual integration of facial and vocal emotion (e.g., [15], but see also 16, for a relevant neuroimaging study). As a limitation, most of these studies used static expressive faces, even though facial motion is known to support emotion recognition (e.g., [17]). Moreover, audio-visual integration typically benefits from temporal synchrony of visual and auditory stimuli, which may be important to attribute stimuli from both modalities to the same underlying event [10]. Evidence from automatic pattern recognition also suggests superior performance when visual and auditory information is integrated at an early featural level [18].

Here we use perceptual adaptation as a tool to investigate bimodal and crossmodal perception of vocal emotion. In general, adaptation to a certain stimulus quality diminishes the response of specific neurons sensitive to that quality, thus enhancing sensitivity to change, and often eliciting “contrastive” aftereffects in perception. For instance, prolonged viewing of a moving adaptor stimulus elicits a prominent motion aftereffect, such that a static stimulus is perceived as moving in a direction opposite to the adaptor [19]. Recently, contrastive adaptation aftereffects were accounted not only for low-level stimulus qualities, but also for complex visual stimuli and faces, among them facial identity [20], face gender [21], facial age [22], or facial expression [21]. In the auditory domain, similar contrastive adaptation aftereffects were more recently reported for the perception of voice gender [23,24], vocal age [25], voice identity [26,27], or vocal affect [28]. 

Of particular relevance for the present study Bestelmeyer et al. [28] presented the first report of auditory adaptation in vocal affect perception. In that study, adaptation to angry vocalization (single /a/-vowels) caused emotion-ambiguous voices (morphed on an angry-fearful continuum) to be perceived as more fearful, and vice versa. A second experiment found equivalent aftereffects for natural and caricatured adaptor voices, which was interpreted to indicate that aftereffects are not exclusively due to low-level adaptation, but rather may depend on higher-level perception of the affective category of the adaptor. While Bestelmeyer et al. [28] studied unimodal voice adaptation only, Fox and Barton [29] investigated the influence of different emotional adaptor types (faces, visual-non-faces, words, and sounds) on facial emotion categorization, using angry-to-fearful facial expression morphs as static test faces. Importantly, while strong and significant aftereffects were elicited by emotional faces, auditory adaptation to emotional sounds did not elicit significant aftereffects. It may be noteworthy that, compared to same-person combinations of adaptor and test faces, adaptor faces from different individuals caused somewhat smaller (though still significant) aftereffects to emotion perception. This could suggest a degree of identity-specific representation of facial expressions. 

The aim of the present study was to extend recent findings [28] of contrastive aftereffects in the perception of vocal affect. Importantly, we compared a condition of unimodal auditory (voice) adaptation with two conditions that have not been studied before. Specifically, we investigated the degree to which bimodal (face-voice) and crossmodal (face) adaptation conditions would also cause aftereffects on the perception of emotion in test voices. A study by Collignon et al. [14] showed audio-visual integration in emotional processing, as fear and disgust categorization was faster and more accurate in bimodal situations as compared to unimodal (auditory or visual) stimuli presentation. Accordingly, we expected bimodal adaptation to elicit larger adaptation effects, when compared to a standard unimodal adaptation condition. In addition, although crossmodal aftereffects of voice-face adaptation have been found to be absent in a study that investigated the perception of facial expressions [29], we considered the possibility that crossmodal face-voice aftereffects might be demonstrated under more favorable conditions, in which both visual and auditory stimuli exhibit a high degree of temporal congruence and represent the same underlying dynamic event. Such conditions should contribute to efficient multisensory processing [10].

In the present study, we therefore co-recorded facial and vocal expressions of emotion, to ensure that visual and auditory representations of the stimuli represented the same underlying events. This allowed us to test the impact of unimodal (auditory), bimodal (audio-visual), and crossmodal (visual only) adaptors on the perception of emotion in the voice. A series of three experiments was conducted which were identical in experimental design, and which only differed in adaptor modality. Note that since “own-gender bias effects” have been previously reported for various aspects of face and voice perception (e.g., [30-32]), we analyzed gender effects at the level of both listeners and experimental stimuli.

## Experiment 1 – Unimodal Adaptation

### Method

#### Ethics Statement

All three experiments in this paper were carried out in accordance with the Declaration of Helsinki, and were approved by the Ethics Committee of the University of Jena. All listeners gave written informed consent and received a payment of € 5 or course credit.

#### Listeners

Twenty-four listeners (12 female) between the ages of 19 and 30 years (*M* = 22.4, *SD* = 2.7) contributed data. None reported hearing disorders. The data of two additional listeners was excluded due to hardware problems.

#### Recording Procedure and Speaker Selection

High-quality audio recordings of four male (mAK, mJN, mSB, mUA) and four female (fDK, fEM, fMV, fSM) native German speakers were obtained in a quiet and semianechoic room using a Sennheiser MD 421-II microphone with a pop protection and a Zoom H4n audio interface (16-bit resolution, 44.1 or 48 kHz sampling rate; upsampled using Adobe Audition to 48 kHz due to synchronization issues). All but one speaker (fSM) were amateur actors. Videos were simultaneously recorded. Among a set of utterances, the relevant ones were four consonant-vocal-consonant-vocal (CVCV) syllables /baka/, /bapa/, /boko/, and /bopo/. After a short and general instruction, we recorded emotional utterances in three blocks in a fixed sequence, starting with neutral and followed by angry and happy conditions. Each utterance was auditioned by the session manager, and repeated several times by the speaker. For the emotional utterances, the session manager first read a short text describing a situation in which people typically react with hot anger or great pleasure, in order to induce angry or happy mood. Each utterance was repeated several times until the session manager was satisfied by the facial and vocal emotion expressed. Speakers were encouraged to make breaks at self-determined point in times. Still water was provided.

To select most convincing emotional utterances, recordings were evaluated by twelve raters (6 female; *M* = 22.7 years, *SD* = 2.2). A total of 282 voice recordings (8 speakers x 4 CVCVs x 3 emotional conditions x 3 repetitions - 6, please note that due to an error in the recording procedure, we did not record /boko/ of male speaker mSB in neutral expression and /bopo/ of female speaker fDK in happy expression) were presented in randomized order and listeners performed a 7-alternative-forced-choice (7-AFC) task with response options for neutral and six basic emotions (angry, happy, sad, disgust, fearful, surprised), and a subsequent rating on perceived intensity of the same stimulus using an 8-point-scale from ‘1 - gar nicht intensiv (neutral)’ to ‘8 – sehr intensiv’ [‘1 – *not intense at all* (*neutral*)’ to ‘8 – very intense’]. For original classification data of emotional stimuli of all eight speakers, please refer to Table S1. 

Several raters stated via questionnaire to know by sight some speakers (mSB, *N* = 6; fSM, *N* = 8; fDK, *N* = 1; fEM, *N* = 1). To avoid interference from familiarity in the perception of emotions, we therefore excluded speakers fSM and mSB. Ratings of voices of the remaining six speakers were in general comparable. Overall, angry stimuli got highest correct classification rates (77%), followed by neutral (67%) and happy (44%). Note that some misclassifications may likely have occurred as a consequence of the experimental design, since listeners explicitly were given seven response options, and thus expected disgust, surprised, and sad stimuli to appear among the utterances. In fact, Table 1 suggests a clear pattern in which, if misclassified, happy utterances tended to be perceived as surprised, and neutral utterances tended to be perceived as sad.

**Table 1:** Classification accuracy (ACC; i.e. proportion correct) and mean intensity ratings for selected stimuli.

Selected Stimuli				Response							
Speaker	Emotion	CVCV	ACC	ANG	DIS	HAP	SUR	NEU	SAD	FEA	miss
fDK	ANG	baka	0.75	9 (5.78)	2 (3.50)	1 (7.00)					
		bapa*	0.92	11 (5.55)	1 (5.00)						
		boko*	0.92	11 (6.27)							1
		bopo	0.92	11 (4.18)	1 (6.00)						
	HAP	baka	1.00			12 (3.83)					
		bapa*	0.92			11 (4.09)				1(6.00)	
		boko*	0.67			8 (4.38)	4 (5.00)				
		bopo1									
	NEU	baka	0.83					10 (1.20)	2 (2.50)		
		bapa*	1.00					12 (1.50)			
		boko*	0.50					6 (1.33)	6 (3.67)		
		bopo	0.58					7 (1.00)	5 (4.40)		
fMV	ANG	baka	0.92	11 (5.91)	1 (6.00)						
		bapa*	0.83	10 (5.40)	1 (8.00)					1 (4.00)	
		boko*	0.67	8 (4.63)	3 (3.00)			1 (1.00)			
		bopo	0.92	11 (5.36)	1 (4.00)						
	HAP	baka	0.58	2 (4.50)		7 (4.00)	3 (5.33)				
		bapa*	0.50	3 (2.67)		6 (4.50)	3 (5.67)				
		boko*	0.42	1 (1.00)	1 (1.00)	5 (6.20)	5 (5.00)				
		bopo	0.58			7 (4.43)	4 (6.00)				1
	NEU	baka	0.75		1 (4.00)			9 (1.78)	2 (3.50)		
		bapa*	0.92					11 (1.64)	1 (2.00)		
		boko*	0.75		1 (2.00)			9 (1.22)	2 (4.50)		
		bopo	0.67			1 (5.00)		8 (1.63)	3 (4.33)		
mAK	ANG	baka	0.58	7 (5.29)		3 (4.33)	2 (4.50)				
		bapa*	0.92	11 (4.00)			1 (5.00)				
		boko*	0.92	11 (4.82)		1 (4.00)					
		bopo	1.00	12 (5.25)							
	HAP	baka	0.58			7 (5.00)	5 (5.00)				
		bapa*	0.83	1 (5.00)		10 (4.80)	1 (8.00)				
		boko*	0.75	2 (3.50)		9 (4.56)	1 (6.00				
		bopo	0.58			7 (5.14)	5 (4.60				
	NEU	baka	1.00					12 (1.42)			
		bapa*	1.00					12 (1.42)			
		boko*	0.92					11 (1.64)	1 (3.00)		
		bopo	0.67	1 (1.00)				8 (1.25)	3 (3.00)		
mUA	ANG	baka	0.92	11 (4.45)	1 (6.00)						
		bapa*	1.00	12 (4.33)			1 (1.00)				
		boko*	0.83	10 (5.20)	1 (4.00)						
		bopo	0.83	11 (4.36)	1 (5.00)						
	HAP	baka	0.50			6 (4.83)	6 (5.33)				
		bapa*	0.67			8 (4.25)	3 (5.33)	1 (1.00)			
		boko*	0.33			4 (4.00)	8 (5.00)				
		bopo	0.33	1 (5.00)	1 (3.00)	4 (5.50)	6 (5.17)				
	NEU	baka	0.92					11 (1.36)	1 (2.00)		
		bapa*	0.83					10 (1.40)	2 (4.00)		
		boko*	0.67					8 (1.13)	4 (4.00)		
		bopo	0.83					10 (1.50)	2 (4.50)		

The number of responses (in total 12; including any misses) and the mean intensity rating (in parentheses, measured on an 8-point scale from “1 - not intense” to “8 - very intense”) is given for each response category, i.e. angry (ANG), disgust (DIS), happy (HAP), surprised (SUR), neutral (NEU), sad (SAD), fearful (FEA). CVCV syllables /baka/ and /bopo/ were used for test stimulus generation, /bapa/ and /boko/ served as adaptor stimuli (marked with an asterisk)^1)^.Note: Due to missing recordings, no ratings were available for /bopo/ utterances by female speaker fDK.

Finally, stimuli of four speakers (fDK, fMV, mAK, mUA) were chosen for the adaptation experiments, based on overall voice classification rates. However, female speaker fMV was selected instead of fEM, because *facial* emotional expression of fEM was judged by the authors and five additional raters to be poor. Stimuli of speaker’s fEM and mJN were used for practice trials.

### Stimuli

#### Preparation

For each utterance (per speaker and emotion), we selected the recording with the highest classification rate among three repetitions. In case of ambiguity, the recording with highest (or, for neutral utterances, lowest) intensity ratings was chosen. The proportion of correct classification for finally selected stimuli was satisfactory (*M* = .767, SEM = .020). Male and female listeners did not differ in their judgments on voices (*ps* ≥ .109), and there were no differences between stimuli used for adaptor and test voices (*ps* ≥ .191). A 3 x 2 ANOVA with factors emotion categories and speaker gender revealed a main effect of emotion, *F*(2,22) = 8.917, *p = .001*, η^2^_p_ =.448, with correct classification rates of .874 ± .029, .625 ± .055, and .802 ± .035, for angry, happy, and neutral stimuli, respectively. There was also a two-way interaction of emotion x speaker gender, *F*(2,22) = 4.823 *p = .018*, η^2^_p_ = .305. No significant differences between speaker genders were observed for both angry and neutral stimuli, *T*s(11) ≤ 1.890, ps ≥ .085. A small difference for happy stimuli of the happy category, *T*(11) = 2.286, *p* = .043 (Ms = .676 ± .061 and .573 ± .058, for female and male, respectively) reflected the fact that male stimuli were slightly more often categorized as “surprised” (see Table 1), a relatively common misclassification that might relate both to the design of the rating, and the fact that no surprised voices were presented. Differences between speaker gender disappeared, *T*(11) = 0.965, *p* = .406, when happy and surprised responses were combined to one category. Overall stimuli of different categories were highly discriminable, with almost no overlap of angry, happy and neutral classifications (see Table 1) and with only small speaker gender differences for happy voices. Classification rates and intensity ratings per stimulus and response category can be found in Table 1. A /bopo/ of fDK in happy intonation, missing from the original recording, was generated by replacing the second /b/ plosive (i.e. duration of closure, plosive release) of a happy /bobo/ recording by a happy /bapa/’s /p/ plosive. The second author and five additional raters could not perceive any modification or peculiarity in the resulting stimulus. Each utterance was saved in a single file (.wav, 48 kHz, mono) and intensity was scaled to 70 dB RMS using Praat [33]. A silence phase of 50 ms was added at the beginning and end of stimuli used to morph test voices. Adaptors in Experiment 1 were voice recordings of /bapa/ and /boko/ in neutral, angry and happy vocal expressions. We added silence phases during 12 video frames (~ 480 ms) both before voice onset and after voice offset. This was done to keep the timing of unimodal adaptors comparable to that of bimodal and crossmodal adaptors (used in Experiments 2 and 3, respectively).

#### Voice Morphing

Test voices were emotion-ambiguous resynthesized voices, resulting from an interpolation of angry and happy CVCVs (/baka/ and /bopo/). We used TANDEM-STRAIGHT [34] based morphing to create test voices with increasing “happy” proportions along the angry-to-happy morph continuum. A test voice of morph level x (MLx) refers to an interpolation between *x*% of the happy and (100-*x*)% of the angry voice recording with *x* ∈ [20,35,50,65,80]. We generated 40 test voices along eight morph continua (4 speakers x 2 CVCVs x 5 ML). Morphing requires manual mapping of corresponding time- and frequency-anchors in the spectrograms. For a more detailed description, please refer to Kawahara et al. [35]. In short, we set time anchors at key features of the utterances (i.e., onset and offset; initial burst of consonants; beginning, middle and end of formant transitions; stable phase of the vowels). We decided to map time anchors in Praat, due to convenient inspection of waveform and spectrogram, and then transferred time anchors to TANDEM-STRAIGHT. At time anchor positions, frequency anchors were then assigned at the center frequency of three to four formants where detectable.

### Design and Procedure

Listeners had to classify 40 emotion-ambiguous test voices along eight morph continua (4 identities x 2 CVCVs x 5 ML), that were presented after adaptation to angry, happy, or neutral vocalizations of two different speakers (both either male or female). To minimize low level adaptation effects, adaptors containing /o/-vowels (/boko/) were combined with test voices containing /a/-vowels (/baka/) and vice versa, i.e. /bapa/ adaptors were combined with /bopo/ test voices. This design produced 240 trials with unique adaptor-test combinations (40 test voices x 2 adaptor speakers x 3 adaptor emotions). Each of the 240 trials was presented twice, resulting in 480 experimental trials. To maximize adaptation effects, these trials were presented in six blocks of 80 trials each, for which adaptor emotion was kept constant. Within each block, trial order was randomized. The order of blocks was counterbalanced across listeners, using a balanced Latin square (e.g., [36]). To summarize, a 3 (adaptor emotion, AEmo) x 2 (test gender, TG) x 5 (morph level, ML) x 2 (adaptor gender, AG) x 2 (listener gender, LG) design was carried out, with both AG and LG as between-subject factors. 

**Figure 1:**
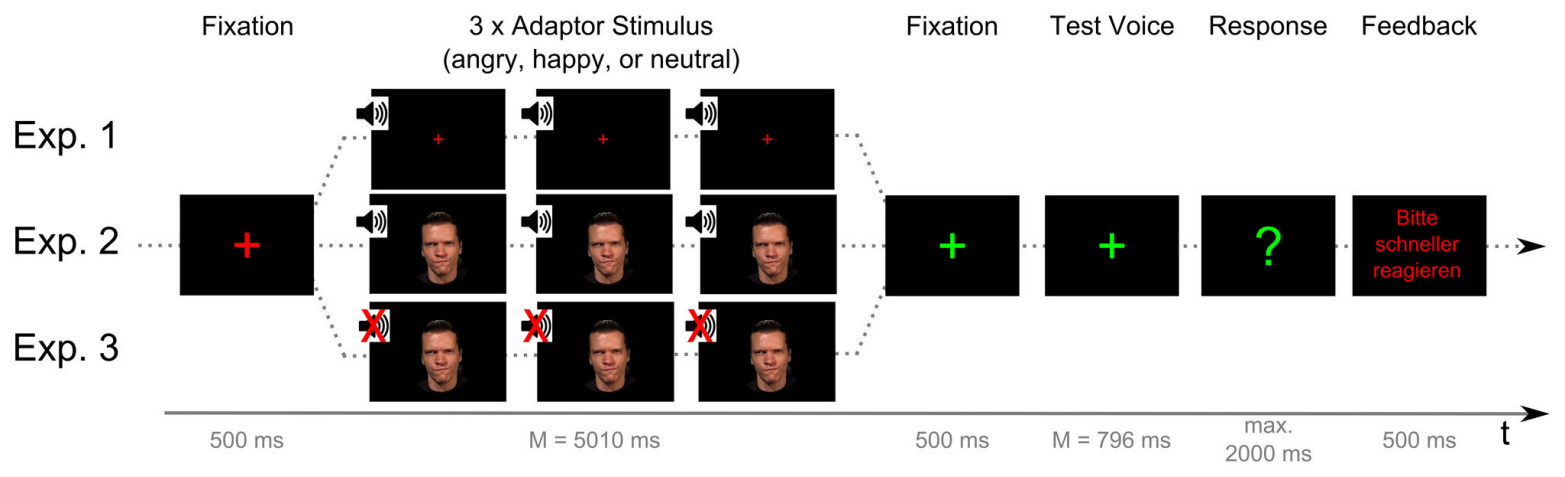
Experimental Trial Design. The general trial design and timing was equivalent in all three Experiments (Exp. 1 – Exp. 3). Experiments differed in adaptor modality only. Note: The person displayed has provided written informed consent for publication of this image.

All instructions were presented in writing on a computer screen, to minimize interference from the experimenter’s voice. After a short practice block consisting of twelve trials with stimuli not used thereafter, listeners had the opportunity to ask questions in case of remaining confusion. Each trial started with a red fixation cross in the center of a black computer screen (500 ms), marking the upcoming adaptor stimulus. The fixation cross remained on the screen while the adaptor stimulus (*M* = 5010 ms, *SD* = 284; consisting of three identical adaptor voices, each with pre- and post-adaptor silence periods of ~480 ms, see Section 2.1.3) was presented. Subsequently, a green fixation cross (500 ms) replaced the red one for 500 ms, to mark the upcoming test voice (*M* = 796 ms ± 37). Listeners were instructed to attentively listen to the adaptor, and to perform an angry-happy 2-AFC classification for the test voice, pressing response keys “k” or “s” on a standard German computer keyboard, respectively. After test voice offset, the green fixation cross was replaced by a green question mark and responses were recorded 2000 ms from stimulus offset. If no response was entered (error of omission) a 500 ms screen prompted for faster response ("Bitte reagieren Sie schneller" ["Please respond faster”]); otherwise, a black screen was shown instead. Each block consisted of 80 randomized trials (2 adaptor voice identities x 2 TG x 2 test voice identities x 2 CVCVs x 5 ML). Individual breaks were allowed after blocks of 40 trials (Figure 1, for general trial design).

### Statistical Analysis

We performed analyses of variance (ANOVAs), using epsilon corrections for heterogeneity of covariances [37] throughout where appropriate. Errors of omission (no key press; 1.05% of all experimental trials) were excluded from the analyses.

### Results and Discussion

An initial 3 x 2 x 5 x 2 x 2 ANOVA on the proportion of happy responses with the factors adaptor emotion (AEmo), test gender (TG), morph level (ML), and between subject factors listener gender (LG) and adaptor gender (AG), did not reveal any effects or interactions involving LG (all ps ≥ .086). We therefore performed an equivalent ANOVA, but without factor listener gender (for a summary of effects, refer to Table 2).

**Table 2:** Summary of ANOVA results from Experiments 1 and 2.

	**Experiment 1**							**Experiment 2**						
**Analyzed**	**Effect**	**df1,df2**	***F***	***p***	***η_p_^2^***	***ε_HF_***		**Effect**		**df1,df2**	***F***	***p***	***η_p_^2^***	***ε_HF_***
**All data**	AEmo	2,44	12.785	< .001***	.368			AEmo		2,44	33.027	< .001***	.600	
	ML	4,88	162.347	< .001***	.881	.561		ML		4,88	84.197	< .001***	.793	.457
	AEmo*TG	2,44	5.280	.009**	.194			AEmo*AG		2,44	4.793	.013*	.179	
	AEmo*TG*AG	2,44	4.136	.023*	.158			AEmo*TG*AG		2,44	8.701	.001**	.283	
	AEmo*ML*AG	8,176	2.446	.016*	.100									
	AEmo*TG*ML*AG	8,176	2.115	.037*	.088									
**AG = m**	AEmo	2,22	8.467	.002**	.435			AEmo		2,22	24.422	< .001***	.689	
	ML	4,44	96.779	< .001***	.898	.574								
	AEmo*TG	2,22	0.784	.469	.067			AEmo*TG		2,22	1.279	.298	.104	
	AEmo*ML	8,88	0.575	.796	.050									
	AEmo*TG*ML	8,88	1.133	.349	.093									
**AG = f**	AEmo	2,22	6.546	.006**	.373			AEmo		2,22	9.759	.001**	.470	
	ML	4,44	68.649	< .001***	.862	.539								
	AEmo*TG	2,22	11.023	< .001***	.501			AEmo*TG		2,22	10.293	.003**	.483	.740
	AEmo*ML	8,88	2.548	.015*	.188									
	AEmo*TG*ML	8,88	2.302	.027*	.173									
**AG = f**	AEmo	2,22	13.531	< .001***	.552			AEmo		2,22	14.350	< .001***	.566	
	**TG = f**	ML	4,44	57.170	< .001***	.839								
	AEmo*ML	8,88	2.332	.025*	.175									
**AG = f**	AEmo	2,22	1.085	.355	.090			AEmo		2,22	.611	.552	.053	
**TG = m**	ML	4,44	23.916	< .001***	.685	.589								
	AEmo*ML	8,88	2.523	.016*	.187									

Summary of results from the overall ANOVAs on the proportion of “happy-responses” with the factors adaptor emotion (AEmo, 3), test gender (TG, 2), morph level (ML 5), and between subject factor and adaptor gender (AG, 2), as well as summary of results of post-hoc ANOVAs performed to follow-up significant interactions of Experiments 1 and 2. Note: Epsilon corrections (*ε*_*HF*_) for heterogeneity of covariances are given where appropriate. Asterisks mark level of significance, ***p < .001, **p < .01, *p < .05, ^*†*^ *p* < .1.

The prominent main effect of ML, *F*(4,88) = 162.347, *p* < .001, *ε*_*HF*_ = .561, *η*_*p*_^2^ = .881, validated the general morphing procedure, as the proportion of happy responses increased with increasing morph level (Ms = .322 ± .023, .426 ± .030, .514 ± .028, .612 ± .023, and .694 ± .020, for ML20 to ML80, respectively). This observation is further confirmed by a strong linear trend in polynomial contrast analysis, *F*(1,22) = 288.341, *p* < .001, *η*_*p*_^2^ = .929. Importantly, the main effect of AEmo was significant, *F*(2,44) = 12.785, *p* < .001, *η*_*p*_^2^ = .368. In line with our hypothesis, test voices were perceived as more happy after prior adaptation to angry voices, and less happy after prior adaptation to happy voices (Ms = .567 ± .025 and .472 ± .025, respectively), with the neutral adaptation condition producing intermediate “happy” classification rates close to chance level (*M* = .502 ± .026). 

These main effects were further qualified by several interactions involving AG and TG (refer to Table 2 for details). To disentangle the four-way interaction of AEmo x TG x ML x AG, *F*(8,176) = 2.115, *p* = .037, *η*_*p*_^2^ = .088, we computed two separate 3 x 2 x 5 ANOVAs for male and female adaptor conditions, with factors AEmo, TG, and ML.

**Figure 2:**
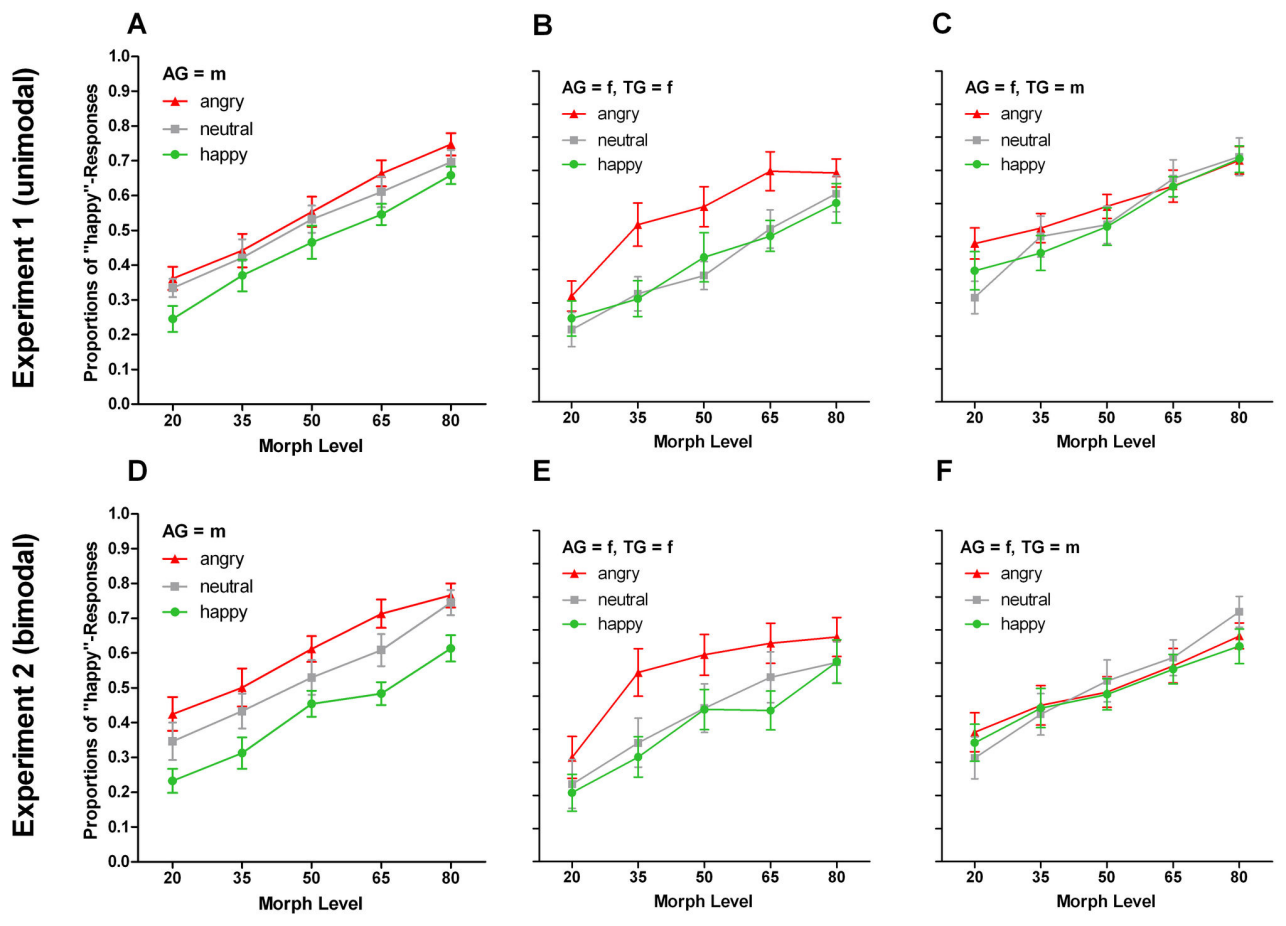
Adaptation-induced Aftereffects and Effects of Adaptor Gender and Test Voice Gender in Experiments 1 and 2. Mean proportions of “happy”-responses to morphed test voices in Experiment 1 (unimodal adaptation, A-C) and Experiment 2 (bimodal adaptation, D-F), depending on morph level and adaptor emotion. (A, D) Male adaptation condition, collapsed across test voice gender. (B, E) Female adaptation condition, with female test voices. (C, F) Female adaptation condition, with male test voices.

For male adaptors, both main effects of ML and AEmo survived (Figure 2A). With respect to adaptation aftereffects, happy (*M* = .457 ± .034) differed from both neutral and angry adaptation conditions (Ms = .519 ± .036, and .554 ± .034, respectively), *T*(11) = 2.904, *p* = .014, and *T*(11) = 3.461, *p* = .005, respectively. The difference between angry and neutral adaptation conditions was not significant, *T*(11) = 1.612, *p* = .135. None of the above mentioned interactions that had involved AG were significant when considering male adaptors only (ps ≥ .349).

For female adaptors, while both main effects of ML and AEmo also survived, they were qualified by the three-way interaction AEmo x TG x ML, *F*(8,88) = 2.302, *p* = .027, *η*_*p*_^2^ = .173. A separate 3 x 5 ANOVA with factors AEmo and ML for female test voices (Figure 2B) yielded an interaction AEmo x ML, *F*(8,88) = 2.332, *p* = .025, *η*_*p*_^2^ = .175, which reflected significant differences between AEmo at the more emotion-ambiguous morph levels ML35 to ML65, *F*s(2,22) ≥ 7.577, ps ≤ .003, *η*_*p*_^2^ ≥ .408, but not at ML20 and ML80 (*ps* = .064 and .181, respectively). Pair wise comparison between adaptation conditions at ML35 to ML65 revealed significant differences between angry and happy adaptation, all *T*s(11) ≥ 2.711, ps ≤ .020, and again between angry and neutral adaptation, *T*s(11) ≥ 3.803, ps ≤ .003. No differences were seen between neutral and happy adaptation (*ps* ≥ .379). For male test voices (Figure 2C) the interaction AEmo x ML, *F*(8,88) = 2.523, *p* = .016, *η*_*p*_^2^ = .187, was also significant, but a significant difference between adaptation conditions was found at ML20 only, *F*(2,22) = 5.762, *p* =.010, *η*_*p*_^2^ = .344, and not at any other ML (ps ≥ .243). At ML20, differences were significant between angry and happy adaptation, *T*(11) = 2.337, *p* = .039, and between angry and neutral adaptation, *T*(11) = 3.283, *p* = .007, but not between neutral and happy adaptation (*p* = .183).

Overall, the results of Experiment 1 corroborate and extend recent reports of high-level aftereffects of adaptation to vocal expression [28]. We found that emotion-ambiguous voices (on an angry-to-happy continuum) were perceived as more happy after adaptation to angry voices, and as more angry after adaptation to happy voices. Although these effects were independent of listener gender, more subtle modulations of adaptation aftereffects were caused by adaptor voice gender and other experimental variables. A more detailed discussion of these findings will be provided in the general discussion.

## Experiment 2 – Bimodal Adaptation

### 2.1: Method

#### 2.1.1: Listeners

Twenty-four new listeners (12 female) between the ages of 18 and 35 years (*M* = 22.8, *SD* = 3.9) contributed data. None reported hearing disorders. Procedures of informed consent, payment, and ethical approval were as in Experiment 1. The data of four additional listeners were not analyzed due to a programming error.

#### 2.1.2: Stimuli

Test stimuli were the same 40 synthesized test voices as used in Experiment 1 (see Section 1.1.4). Adaptor stimuli were video recordings that had been captured simultaneously to the voice recordings and that were synchronized with the auditory adaptor stimuli of Experiment 1. Videos displayed the same four speakers while articulating /bapa/ and /boko/ in angry, happy, or neutral expression.

#### 2.1.3: Design and Procedure

Design and procedure were as in Experiment 1 (see Section 1.1.5), with the only difference that adaptors were bimodal videos. 

#### 2.1.4: Statistical Analysis

Statistical analyses were performed in analogy to Experiment 1. Errors of omission were excluded (omissions averaged to 0.48% of experimental trials).

### 2.2: Results and Discussion

As an initial ANOVA on the proportion of happy responses with the same factors as in Experiment 1 again did not reveal any effects or interactions involving listener gender (all ps ≥ .062), we performed an equivalent ANOVA without listener gender (for a summary of effects, refer to Table 2). Unsurprisingly, a strong main effect of ML, *F*(4,88) = 84.197, *p* < .001, *ε*_*HF*_ = .457, *η*_*p*_^2^ = .793, with a prominent linear trend, *F*(1,22) = 111.687, *p* < .001, *η*_*p*_^2^ = .835, was again found. In addition, the ANOVA revealed a prominent main effect of AEmo, *F*(2,44) = 33.027, *p* < .001, *η*_*p*_^2^ = .600, reflecting a contrastive pattern of aftereffects for angry, neutral and happy adaptation conditions (Ms = .577 ± .025, .511 ± .028, and .440 ± .021, respectively), which was further qualified by a two-way interactions with adaptor gender, and an additional three-way interaction involving both adaptor and test gender (Table 2). To investigate the nature of this three-way interaction, we analyzed data separately for each adaptor gender, by means of two separate 3 x 2 ANOVAs with factors AEmo x TG. 

For male adaptors, the main effect of AEmo survived (Figure 2D), whereas the interaction AEmo x TG was not significant (ps ≥ .298). With respect to the main effect of AEmo, all pair-wise comparisons between means of angry, neutral and happy adaptation conditions (Ms = .603 ± .043, .533 ± .048, and .419 ± .033, respectively), were significant |*T*s(11)| ≥ 2.511, ps ≤ .029, with largest differences between angry and happy adaptation, *T*(11) = 8.115, *p* < .001.

For female adaptors, the main effect of AEmo also survived, but was qualified by a significant interaction of AEmo x TG (Table 2). For female test voices alone, an effect of AEmo was significant; pair-wise comparisons between means of angry, neutral and happy adaptation conditions (Ms = .570 ± .044, .443 ± .050, and .409 ± .037, respectively), were significant for both angry compared to neutral and to happy, with *T*(11) ≥ 4.831, *p* = .001, but not for neutral and happy, *T*(11) = .931, *p* = .372. By contrast, no significant effect of AEmo was observed for male test voices (Figures 2E and 2F, respectively).

Taken together, Experiment 2 demonstrated substantial aftereffects of adaptation to bimodal expressive videos on the perception of vocal emotion. The pattern of observed effects comprised fewer interactions, but was generally similar to the effects of adaptation to unimodal voice adaptors in Experiment 1 (Figures 2A-2C). A visual inspection of the results also suggests that bimodal adaptors were somewhat more efficient than unimodal adaptors in causing aftereffects in vocal emotion perception. The effects of bimodal adaptation were again not significantly modulated by listener gender, but were modulated by adaptor and test voice gender. A more detailed discussion of these findings will be provided in the general discussion.

## Experiment 3 – Crossmodal Adaptation

### 3.1: Method

#### 3.1.1: Listeners

Twenty-four new listeners (12 female) between the ages of 19 and 34 years (*M* = 23.7, *SD* = 3.7) contributed data. None reported hearing disorders. Data from four additional listeners were not analyzed due to hardware or software problems. Procedures of informed consent, payment, and ethical approval were as in Experiments 1 and 2.

#### 3.1.2: Stimuli

Test stimuli were the same 40 synthesized test voices as used in Experiments 1 and 2 (see Section 1.1.4). Adaptor stimuli were video as used in Experiment 2, but this time presented without sound, i.e. participants adapted to silently articulating emotional face videos.

#### 3.1.3: Design and Procedure

Design and procedure were the same as in Experiment 1, with the only difference that adaptors were silently articulating videos (crossmodal adaptation).

#### 3.1.4: Statistical Analysis

Statistical analyses were performed in analogy to Experiments 1 and 2. Errors of omission (in total 1.17% of all experimental trials) were excluded.

### 3.3: Results and Discussion

The initial ANOVA on the proportion of happy responses, with the same factors as in Experiments 1 and 2, revealed a main effect of ML, *F*(4,80) = 147.740, *p* < .001, *ε*_*HF*_ = .701, *η*_*p*_^2^ = .881 (with Ms = .321 ±.023, .426 ± .027, .527 ± .031, .603 ± .025, .686 ± .026, for ML20 to ML80, respectively). The increase of happy responses per ML was confirmed by a strong linear trend in polynomial contrast analysis, *F*(1,20) = 238.898, *p* = .001, *η*_*p*_^2^ =.923. The main effect of morph level was qualified by an interaction with test gender, *F*(4,80) = 6.038, *p* = .001, *ε*_*HF*_ = .842, *η*_*p*_^2^ = .232. At ML20, female test voices were perceived less happy as compared to male test voices, *T*(23) = -4.589, *p* < .001 (Ms = .241 ± .026, .401 ± .030, respectively). This difference was not significant for any other ML, *|T*s(23)| ≤ 1.271, ps ≥ .216.

**Figure 3:**
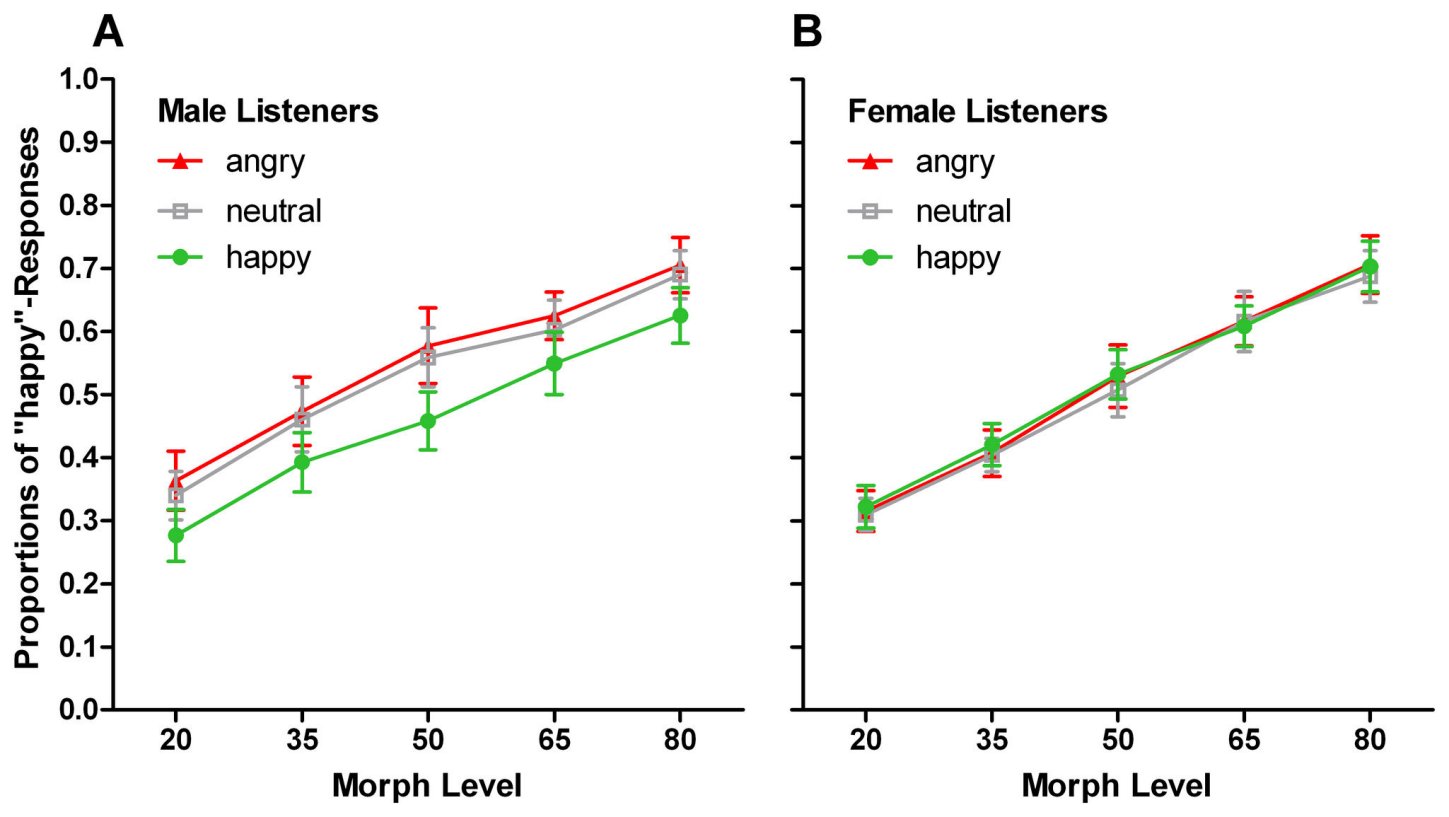
Crossmodal Adaptation-induced Aftereffects and Effects of Listener Gender in Experiment 3. Mean proportions of “happy”-responses to morphed test voices, depending on morph level and adaptor emotion. (A) Male listeners showed crossmodal adaptation effects, whereas (B) female listeners did not.

In contrast to Experiments 1 and 2, the main effect of AEmo was only marginally significant, *F*(2,40) = 2.489, *p* = .096, *η*_*p*_^2^ = .111 (Table 3). Importantly, there was a significant interaction between adaptor emotion and listener gender, *F*(2,40) = 3.277, *p* = .048, *η*_*p*_^2^ = .141. To disentangle role of listener gender, we computed two separate 3 x 2 x 5 x 2 ANOVAs for male and female listeners, with factors AEmo, TG, ML, and AG. For female listeners, we found the expected main effect of ML, but no effect of AEmo, *F*(2,22) = 0.125, *p* = .883, *η*_*p*_^2^ = .011 (Figure 3B). For male listeners, analysis revealed a significant main effect of AEmo, *F*(2,22) = 5.724, *p* = .010, *η*_*p*_^2^ = .342 (Figure 3A). With respect to adaptation aftereffects, happy (*M* = .460 ± .042) differed from both neutral and angry adaptation conditions (Ms = .530 ± .039 and .549 ± .044, respectively; *T*(11) = -2.641, *p* = .023, *T*(11) = 2.904, *p* = .014, respectively). The difference between angry and neutral adaptation condition was not significant, *T*(11) = 0.721, *p* = .486. 

**Table 3:** Summary of ANOVA results from Experiment 3.

Analyzed	Effect	df1, df2	*F*	*p*	*η_p_^2^*	*ε_HF_*
Both LGs	AEmo	2,40	2.489	.096†	.111	
	ML	4,80	147.740	< .001***	.881	.701
	TG x ML	4,80	6.038	.001**	.232	.842
	AEmo x LG	2,40	3.277	.048*	.141	
LG = male	AEmo	2,22	5.724	.010**	.342	
	ML	4,44	63.646	<.001***	.853	.674
	TG x ML	4,88	4.766	.003**	.302	
LG = female	AEmo	2,22	0.125	.883	.011	
	ML	4,44	87.071	<.001***	.888	.422
	TG x ML	4,88	2.376	.095†	.178	.683

Summary of results from the ANOVAs on the proportion of “happy”-responses with the factors adaptor emotion (AEmo, 3), test gender (TG, 2), morph level (ML, 5), and between subject factors listener gender (LG, 2) and adaptor gender (AG, 2), as well as a summary of results of post-hoc ANOVAs performed per listener gender. Note: Epsilon corrections (*ε*_*HF*_) for heterogeneity of covariances are given where appropriate. Asterisks mark level of significance, ***p < .001, **p < .01, *p < .05, ^*†*^ *p* < .1.

In contrast to Experiments 1 and 2, Experiment 3 revealed differences between female and male listeners. Specifically, whereas female participants did not show any crossmodal adaptation effect, substantial aftereffects from adaptation to crossmodal silent videos were found in male participant s. A more detailed discussion of these findings will be provided in the general discussion.

## Comparison of Adaptation Effects between Experiments

### 4.1: Statistical Analysis

In order to directly compare aftereffects across the three experiments, we calculated the magnitude of adaptation aftereffects for each experimental condition, by subtracting the proportions of happy responses in the happy adaptation condition from the proportions of happy responses in the angry adaptation condition. We then computed a 2 x 5 x 2 x 3 x 2 ANOVA with factors test gender (TG) and morph level (ML) as within subject factors, and adaptor gender (AG), adaptor modality (AMod; unimodal, bimodal, and crossmodal, corresponding to Experiments 1, 2, and 3), and listener gender (LG) as between subject factors.

### 4.2: Results and Discussion

For a summary of effects, refer to Table 4. There was a prominent main effect of adaptor modality, *F*(2,60) = 5.880, *p* = .005, *η*_*p*_^2^ = .164. Pair wise comparisons suggested that adaptation effects in the bimodal condition (*M* = 13.672% ± 1.770) were significantly larger than those in the crossmodal condition (*M* = 4.293% ± 2.336), *T*(46) = 3.199, *p* = .002. The numerical difference between bi- and unimodal condition (*M* = 9.560% ± 1.844) failed to reach significance, *T*(46) = 1.609, *p* = .115, as did the difference between uni- and crossmodal conditions, *T*(46) = 1.770, *p* = .083. 

**Figure 4:**
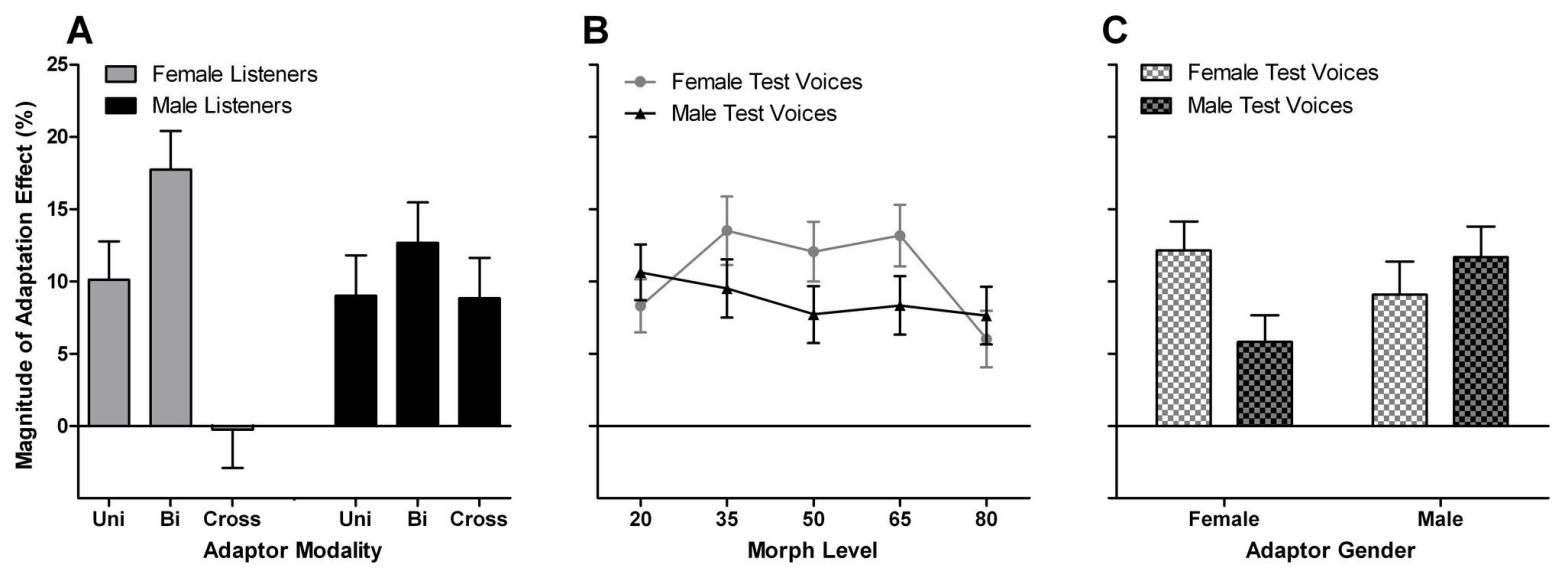
The Magnitude of Adaptation Effects in Experiments 1 to 3. (A) The interaction of adaptor modality and listener gender shows that female listeners exhibited numerically enhanced bimodal adaptation effects, and no crossmodal adaptation effects; for male listeners, adaptation effects were similar across adaptor modalities. (B) The interaction of test voice gender and morph level reflects larger adaptation effects for more ambiguous morph levels, specifically for female test voices. (C) The interaction of adaptor gender and test voice gender reflects larger adaptation effects for gender-congruent adaptor-test combinations for female adaptors, a pattern that was clear for Experiments 1 and 2, but not for Experiment 3. Note: The magnitude of adaptation effects was calculated by subtracting the percentages of “happy”-responses in the happy adaptation condition from the percentages of “happy”-responses in the angry adaptation condition.

There was, however, a strong trend for an interaction of adaptor modality and listener gender, *F*(2,60) = 3.147, *p* = .050, *η*_*p*_^2^ = .095 (Figure 4A). No further interactions involving LG approached significance. We computed two ANOVAs in order to evaluate effects of adaptor modality, separately for female and male listeners. For *female* listeners, the magnitude of adaptation effects differed significantly between adaptor modalities, *F*(2,36) = 9.089, *p* = .001, *η*_*p*_^2^ = .355. Crossmodal adaptation effects (*M* = -0.253% ± 3.131) did not differ significantly from zero, *T*(11) = -.081, *p* = .937, whereas both uni- and bimodal adaptation effects did, *T*s(11) ≥ 4.495, *p*s < .001. Pairwise comparisons revealed significant differences between uni- and crossmodal, *T*(22) = 2.688, *p* = .013, and between bi- and crossmodal adaptation, *T*(22) = 3.920, *p* = .001. There was only a numerical tendency for bimodal adaptation effects to be greater than unimodal adaptation effects, *T*(22) = 1.622, *p* = .119 (Ms = 15.674% ± 2.589, and 10.111% ± 2.250, respectively). By contrast, for *male* listeners, the magnitude of adaptation effects was more uniform, averaging around 10%, and did not differ significantly between adaptor modalities, *F*(2,36) = 0.314, *p* = .732, *η*_*p*_^2^ = .019. Moreover, adaptation effects for male listeners were greater than zero for all modalities, *T*s(11) ≥ 2.904, ps ≤ .014, with Ms = 8.840 % ± 3.044, 9.009% ± 3.016, and 11.670% ± 2.383, respectively for cross-, uni-, and bimodal adaptation conditions.

**Table 4:** Summary of ANOVA results comparing magnitude of adaptation effect between all three experiments.

Analyzed	Effect	df1, df2	*F*	*p*	*η_p_^2^*	*ε_HF_*
Both LGs	AEmo	2,40	2.489	.096^†^	.111	
	ML	4,80	147.740	<.001***	.881	.701
	TG x ML	4,80	6.038	.001**	.232	.842
	AEmo x LG	2,40	3.277	.048*	.141	
LG = male	AEmo	2,22	5.724	.010**	.342	
	ML	4,44	63.646	<.001***	.853	.674
	TG x ML	4,88	4.766	.003**	.302	
LG = female	AEmo	0.125	2,22	.883	.011	
	ML	4,44	87.071	<.001***	.888	.422
	TG x ML	4,88	2.376	.095^†^	.178	.683

Summary of results from the ANOVAs on the magnitude of the adaptation effect, computed as difference between angry and happy adaptation condition with the factors test gender (TG, 2), morph level (ML, 5), and between subject factors adaptor gender (AG, 2), adaptor modality (AMod, 3), and listener gender (LG, 2). Note: Epsilon corrections (*ε*_*HF*_) for heterogeneity of covariances are given where appropriate. Asterisks mark level of significance, ***p < .001, **p < .01, *p < .05, ^*†*^ *p* < .1.

There was also a main effect of TG, *F*(1,60) = 4.838, *p* = .032, *η*_*p*_^2^ = .075, that was qualified by several interactions (Table 4). The interaction TG x AG interaction (Figure 4C) suggested larger adaptation effects for same-gender adaptor- and test-stimulus combinations, but was further qualified by adaptor modality, TG x AG x AMod, *F*(2,60) = 3.623, *p* = .033, *η*_*p*_^2^ = .108. We computed separate 2 x 3 ANOVAs per adaptor gender, with factors *gender congruency* (congruent: adaptor and test of the same gender; incongruent: adaptor and test of different gender) and adaptor modality. For female adaptation conditions, a significant main effect of gender congruency, *F*(1,33) = 24.547, *p* = .001, *η*_*p*_^2^ = .427, was qualified by an interaction with adaptor modality, *F*(1,33) = 5.646, *p* = .008, *η*_*p*_^2^ = .244. Differences between congruent and incongruent adaptor-test-voice-combinations were found in uni- and bimodal female adaptation condition, *Ts*(11) ≥ 3.634, ps ≤ .004, but not in crossmodal adaptation condition, *T*(11) = 0.258, *p* = .801. For male adaptation condition, no effects of congruency were found (*ps* ≥ .325). Note that half of the same-gender trials involved same-identity combinations of adaptor and test. Therefore, to test whether gender congruency effects were in fact caused by identity congruency [29], we analyzed the effect of identity congruency for same-gender trials. A 2 x 4 x 2 x 3 ANOVA on the magnitude of adaptation effect with factors *identity congruency* (congruent adaptor and test of the same identity; incongruent: adaptor and test of different identity), adaptor identity (4, fDK, fMV, mAK, mUA) and between subject factors adaptor gender (AG) and adaptor modality (AMod). Importantly, there was no indication for greater aftereffects following identity congruent trials as compared to identity incongruent trials, *Ms* = 11.3% ± .013 and 12.5% ± .013, respectively; *F*(1,132) = 0.540, *p* =.464, *η*_*p*_^2^ = .004. There was also no significant interaction involving identity congruency. Separate analyses per AG revealed no consistent effect of identity congruency, for both male and female adaptor identities, |*T*s(35)| < 1.594, *p*s >. 119.

Further results from the ANOVA across experiments are briefly reported for the sake of completion, although these confirmed in parts findings from the analyses of individual experiments, and did not interact with adaptor modality. An interaction of ML x TG, *F*(4,240) = 2.781, *p* = .027, *η*_*p*_^2^ = .044, revealed that increased aftereffects at more ambiguous morph levels were found for female test voices, *F*(4,284) = 3.801, *p* = .006, *ε*_*HF*_ = .928, *η*_*p*_^2^ = .051, but not for male test voices, *F*(4,284) = 0.501, *p* = .735, *η*_*p*_^2^ = .007 (Figure 4B).

In sum, the magnitude of adaptation effects on the perception of vocal emotion (computed as differences between angry and happy adaptation conditions), showed a different pattern between female and male listeners. In male listeners, we found a similar adaptation effect across adaptor modalities of ~10%. By contrast, in female listeners, there was no adaptation effect at all in crossmodal (silent video) adaptation condition, whereas bimodal adaptation tended to elicit larger effects compared to unimodal adaptation (although the latter difference was not significant, possibly due to limited statistical power as a result of the between-subjects design).

## General Discussion

To probe the multimodal nature of emotion perception, we conducted a series of three experiments that assessed the influence of perceptual adaptation to different adaptor modalities on the perception of vocal emotional expressions. We used unimodal voices adaptors (Experiment 1), bimodal face-voice video adaptors (Experiment 2), or the same video adaptors without sound as crossmodal adaptors (Experiment 3). 

We demonstrated contrastive aftereffects of adaptation to happy or angry voices, such that test voices morphed on a happy-to-angry continuum were perceived as more happy after prior adaptation to angry voices, and vice versa. These results confirm and extend those by Bestelmeyer et al. [28], who had first reported similar effects using tokens of the vowel /a/ that were morphed on an angry-to-fearful continuum. Our novel findings of crossmodal aftereffects (although only clear for male listeners) may be seen at variance with earlier studies [29] that did not find evidence for crossmodal aftereffects in emotion perception in a face perception task. One possible reason for the present successful demonstration of crossmodal aftereffects could be that we used dynamic video adaptors which not only represented emotional expressions, but which actually represented equivalent underlying actions as the unimodal voice adaptors did. This idea considers that crossmodal processing depends on both congruent temporal information [10] and on higher level factors such as audio-visual stimulus congruency, both of which may contribute to the “unity assumption” [38]. Note that the present data alone do not exclude the possibility that crossmodal adaptation in emotion perception could be unidirectional, particularly when considering that Fox and Barton [29] did not find an effect of emotional auditory adaptors on static face perception (but see 12 for the explicit suggestion of mandatory bidirectional links between faces and voices in emotion perception). Thus, further research is required to determine whether crossmodal adaptation by voice adaptors on the perception of dynamic facial emotions can be demonstrated. 

Another observation, although the relevant effect failed to reach statistical significance, was that bimodal adaptors elicited numerically larger aftereffects on vocal emotion perception when compared to unimodal adaptors. At a broad level, such a finding could be in line with the idea that emotional expressions from faces and voices are processed in a multimodal manner [5,6]. We also note that not only was the impact of adaptor modality strong for female but not male listeners, but also that bimodal adaptation appeared to increase aftereffects somewhat more for female than for male listeners. While this finding clearly requires replication, it might be tentatively related to reports from spoken word perception, according to which women more efficiently integrate emotional information from prosodic and semantic sources, compared to men [39,40]. Although the vast literature on emotion processing is often taken to suggest that women more effectively process emotional signals, and may tend to show more empathy-related responses [41], it has also been proposed that emotional signals provide more behaviorally relevant cues for men [42], and that men might be more efficient in emotion regulation in some conditions [43]. Those extensive reviews of sex differences in emotion processing generally indicate differences in the relevant neural networks, but also revealed a host of conflicting results. In the present study, crossmodal adaptation effects on vocal emotion perception were absent in women, while such effects were prominent in men. Although the precise mechanisms underlying this difference remain unclear, one possibility is that women (but not men) depend on simultaneous bimodal stimulation for face-voice processing to occur. 

Finally, irrespective of listener gender, we also obtained some differences related to the gender of adaptor and test stimuli. First, for female test voices, adaptation effects were larger at emotion-ambiguous morph levels, whereas for male test voices adaptation effects were similar across the entire morph continuum (Figure 4B). Second, we observed significantly larger adaptation effects in gender-congruent adaptor-test combinations overall (Figure 4C). We note that this gender-congruency effect on vocal emotion adaptation was observed particularly for female adaptors in Experiments 1 and 2, i.e. when adaptors contained voices. Although original female happy voices (used as adaptors and for test voice morphing) were classified somewhat better than male happy voices (since the latter elicited slightly more “surprise” responses), this difference cannot explain the absence of aftereffects elicited by female adaptors for both unimodal and bimodal conditions in male test voices (Figure 2C and 2F). Moreover, male adaptors elicited similar aftereffects to the same test voices, irrespective of test voice gender. This finding could therefore indicate that acoustic cues conveying emotional expression [1] are not entirely independent of speaker gender, although this conclusion is limited by the small number of voices used in the present study. Note that both gender-specific and gender-independent contributions to aftereffects have previously been described for the perception of age from both faces [22] and voices [27]. Note also that our effects of adaptor-test gender congruency do not appear to relate to an effect of congruency in the identity of adaptor and test speakers, since identity-congruent adaptor-test combinations clearly did not yield larger aftereffects when compared to identity-incongruent combinations. This could be a relevant contrast with a paper on face adaptation by Fox and Barton [29], who found reduced expression aftereffects for identity incongruent adaptor-test combinations. Although the reasons for these different outcomes are not completely clear, we note that while facial identity is easily perceived even from briefly stimuli, the difficulty to perceive voice identity from brief auditory samples (e.g., [31]), could be a factor for the absence of identity congruency effects in the present study.

While the present study has revealed a number of novel and clear findings, several limitations should also be noted. First, because our study involved a limited number of speakers, utterance types, and emotional expressions, it remains to be determined whether our results generalize to other situations. We note, however, that one other study reported similar unimodal vocal emotion aftereffects for angry-to-fearful test voice continua, and with only /a/ vowel utterances [28]. A degree of variability in our results might also be attributed to stimulus properties, such as differences in emotional expressiveness of individual stimuli. For instance, not all raters perceived emotionally “neutral” stimuli as “neutral” (Table 2), and this could have contributed to the finding that the neutral adaptation condition did not always generate classifications that were exactly midway between those generated by adaptation to angry or happy adaptors. However, it should also be kept in mind that these observations could reflect a degree of individual differences between raters, who can often exhibit different “category boundaries”(cf. [21], for further discussion).

To conclude, the present series of experiments confirms recent findings of contrastive aftereffects in vocal emotion perception caused by adaptation. Here we provide the first evidence for crossmodal aftereffects in emotion perception, elicited by silent videos showing dynamic facial expressions of equivalent emotional events. Overall, our results pose strong support for the idea that the perception of emotions is multimodal in nature. Moreover, we also observed prominent gender differences which are attributed to crossmodal processing, and possibly to bimodal face-voice integration, and we suggest that both aspects warrant further research.

## Supporting Information

Table S1 **Classification data of emotional stimuli of eight speakers in the rating experiment.** Classification data (percentages) for the angry, happy and neutral voice recordings of eight speakers (4 female), and mean classification accuracy (ACC). Speakers fSM and mSB were excluded due to listener reports on familiarity. Speakers fDK, fMV, mAK, mUA were chosen for the adaptation experiments. Note: Percentages marked with an asterisk are based on = 108 ratings, for all others, *N* = 144.
(DOCX)
