# Supplementary material for: Adaptation Aftereffects in Vocal Emotion Perception Elicited by Expressive Faces and Voices
Source: PLoS One. 2013 Nov 13;8(11):e81691. doi: 10.1371/journal.pone.0081691 (PMC3827484; doi:10.1371/journal.pone.0081691)
Supplement: Table S1 — Classification data of emotional stimuli of eight speakers in the rating experiment. Classification data (percentages) for the angry, happy and neutral voice recordings of eight speakers (4 female), and mean classification accuracy (ACC). Speakers fSM and mSB were excluded due to listener reports on familiarity. Speakers fDK, fMV, mAK, mUA were chosen for the adaptation experiments. Note: Percentages marked with an asterisk are based on = 108 ratings, for all others, N = 144. (DOCX) [file pone.0081691.s001.docx]

Table S1. Classification data of emotional stimuli of eight speakers in the rating experiment.

| Intended emotion: |  | angry | | | |  | happy | | | |  | neutral | | | |  |
| --- | --- | --- | --- | --- | --- | --- | --- | --- | --- | --- | --- | --- | --- | --- | --- | --- |
| Perceived emotion: |  | angry | disgust | others | missed |  | happy | surprised | others | missed |  | neutral | sad | others | missed | ACC |
| fDK |  | 83.33 | 7.64 | 7.64 | 1.39 |  | 68.52* | 25.93* | 4.63* | 0.93* |  | 59.72 | 39.58 | 0.69 | 0.00 | 70.52 |
| fEM |  | 77.78 | 11.11 | 9.03 | 2.08 |  | 34.03 | 50.69 | 15.28 | 0.00 |  | 60.42 | 37.50 | 1.39 | 0.69 | 57.41 |
| fMV |  | 72.92 | 23.61 | 3.47 | 0.00 |  | 41.67 | 40.97 | 15.28 | 2.08 |  | 69.44 | 25.69 | 4.86 | 0.00 | 61.34 |
| fSM |  | 96.53 | 2.08 | 1.39 | 0.00 |  | 36.81 | 45.83 | 16.67 | 0.69 |  | 69.44 | 26.39 | 4.17 | 0.00 | 67.59 |
| mAK |  | 79.17 | 3.47 | 17.36 | 0.00 |  | 46.53 | 31.94 | 21.53 | 0.00 |  | 79.86 | 14.58 | 5.56 | 0.00 | 68.52 |
| mUA |  | 81.25 | 9.72 | 8.33 | 0.69 |  | 30.56 | 64.58 | 4.17 | 0.69 |  | 68.06 | 30.56 | 1.39 | 0.00 | 59.95 |
| mJN |  | 65.28 | 21.53 | 12.50 | 0.69 |  | 45.14 | 42.36 | 11.81 | 0.69 |  | 65.28 | 32.64 | 2.08 | 0.00 | 58.56 |
| mSB |  | 86.81 | 7.64 | 5.56 | 0.00 |  | 31.25 | 64.58 | 3.47 | 0.69 |  | 75.00* | 24.07* | 0.93* | 0.00* | 64.35 |
| *M* |  | 80.38 | 10.85 | 8.16 | 0.61 |  | 41.81 | 45.86 | 11.61 | 0.72 |  | 68.40, | 28.88 | 2.63 | 0.09 | 63.53 |
| *SD* |  | 9.95 | 8.35 | 5.46 | 0.77 |  | 6.52 | 12.23 | 6.65 | 0.69 |  | 6.32 | 7.29 | 1.90 | 0.26 | 4.38 |

Classification data (percentages) for the angry, happy and neutral voice recordings of eight speakers (4 female), and mean classification accuracy (ACC). Speakers fSM and mSB were excluded due to listener reports on familiarity. Speakers fDK, fMV, mAK, mUA were chosen for the adaptation experiments. Note: Percentages marked with an asterisk are based on N = 108 ratings, for all others, N = 144.
